# Supplementary material for: SARS-CoV-2 booster vaccine dose significantly extends humoral immune response half-life beyond the primary series
Source: Sci Rep. 2024 Apr 18;14:8426. doi: 10.1038/s41598-024-58811-3 (PMC11026522; doi:10.1038/s41598-024-58811-3)
Supplement: Supplementary file 1 — Supplementary Information. [file 41598_2024_58811_MOESM1_ESM.pdf]

SARS-CoV-2 booster vaccine dose significantly extends humoral immune response half-life beyond the primary series

March 23, 2024

Chapin S. Korosec, David W. Dick, Iain R. Moyles, James Watmough

Corresponding author emails: chapinSkorosec@gmail.com, dwdick@yorku.ca

Contents

S1 Comorbidity breakdown 2

S2 Observation, error models, and Lasso regression details 3

S3 Population and individual model fits 5

S4 Exploring the age cutoff for statistical comparisons 7

S5 Multivariate statistical analysis 8

S1 Comorbidity breakdown

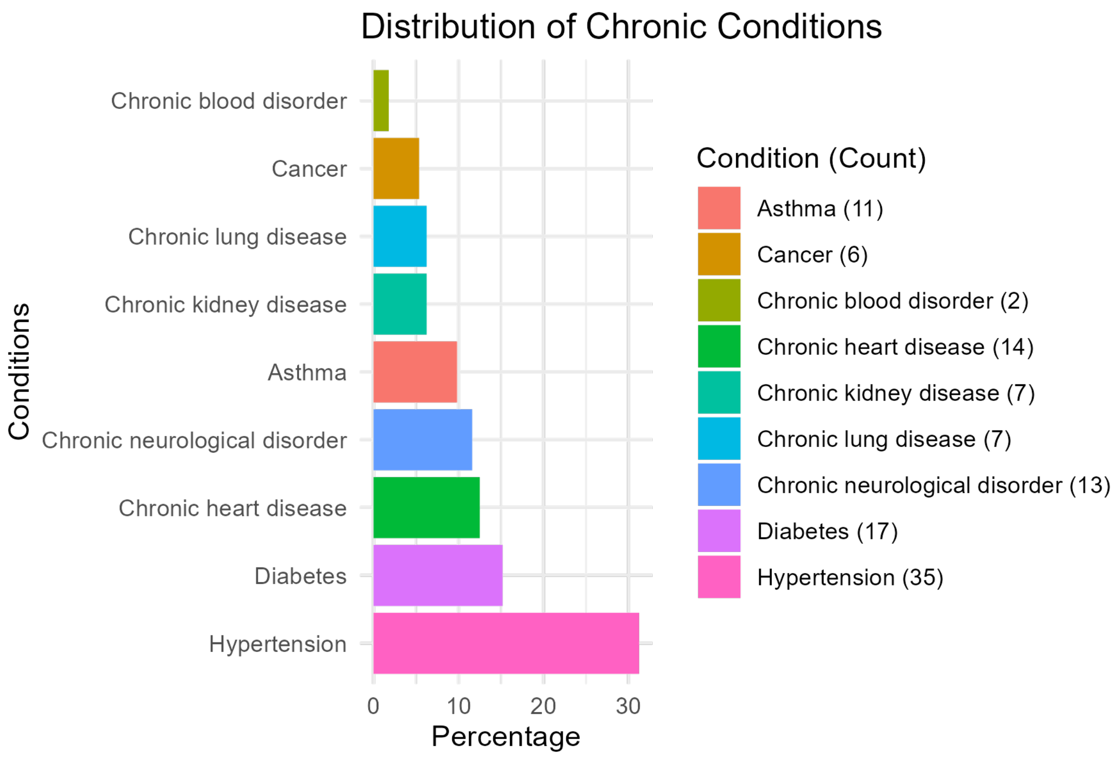

Figure S1: Distribution of chronic conditions among individuals. The bars represent the percentage of diagnoses. The legend details the count of each condition.

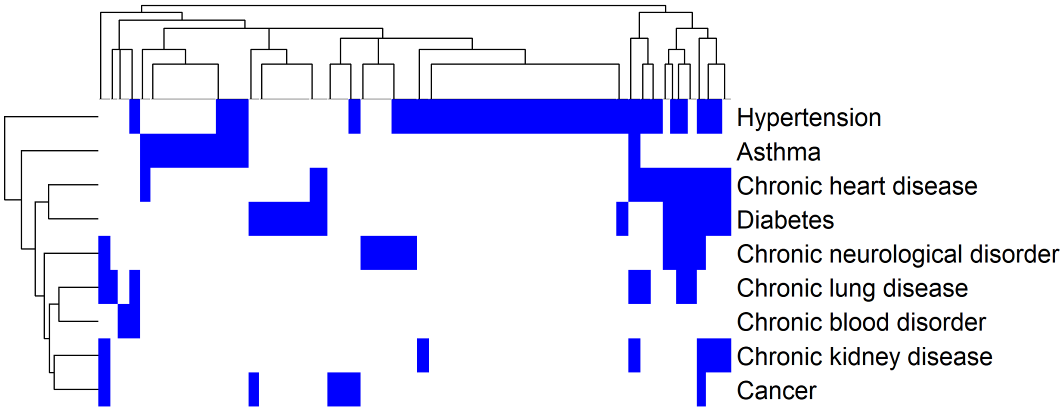

Figure S2: Clustered heatmap illustrating the co-occurrence patterns of chronic comorbidities. Blue cells represent the presence of a chronic comorbidity, with dendrograms showcasing hierarchical clustering based on chronic comorbidity co-occurrence. The counts for each comorbidity are as follows: Hypertension (248), Diabetes (107), Chronic heart disease (88), Asthma (81), Chronic neurological disorder (75), Chronic lung disease (49), Chronic kidney disease (47), Cancer (43), and Chronic blood disorder (15).

S2 Observation, error models, and Lasso regression details

Eq. 1 is fit to individual Anti-S trajectories in Monolix. The observation model used in these fits is given by,

$$y_2 = \log(A) + (a + b\log(A)) * e, \tag{S1}$$

where a and b are error model parameters, and e is a sequence of independent random variables normally distributed with mean 0 and variance 1. The individual model used to fit the decay rate, $\gamma_{j,i}$, is given by,

$$\log(\gamma_{j,i}) = \log(\gamma_{jpop}) + \eta_{\gamma_{j,i}}, \tag{S2}$$

where $\eta_{\gamma_{j,i}}$ are the random effects for the decay rate of the jth dose for the ith individual.

Lasso Regression

Our MVLR model was developed in `R version 4.2.2 (2022-10-31 ucrt)` on a `x86_64-w64-mingw32/x64 (64-bit)` platform, running under `Windows 10 x64 (build 22621)`. For feature selection, Lasso regression was employed using the `glmnet_4.1-7` package [1–4].

Lasso (Least Absolute Shrinkage and Selection Operator) regression is a form of linear regression that introduces a penalty term on the absolute values of the regression coefficients β_j. Specifically, the L1 penalty term is given by:

$$\text{L1 Penalty} = \lambda \sum_{j=1}^{p} |\beta_j|$$

where λ is a non-negative regularization parameter, and β_j represents each coefficient in the linear model.

The L1 penalty encourages sparse solutions by setting some coefficients to zero, effectively performing feature selection.

In R the regression objective function is:

$$\text{Minimize:} \quad \frac{1}{2n} \sum_{i=1}^{n} \left(y_i - \beta_0 - \sum_{j=1}^{p} \beta_j x_{ij} \right)^2 + \lambda \left[(1-\alpha) \frac{1}{2} \sum_{j=1}^{p} \beta_j^2 + \alpha \sum_{j=1}^{p} |\beta_j| \right]$$

Here: - $\frac{1}{2n} \sum_{i=1}^{n} \left(y_i - \beta_0 - \sum_{j=1}^{p} \beta_j x_{ij} \right)^2$ is the loss term, which measures how well the model fits the data. - $\lambda \left[(1-\alpha) \frac{1}{2} \sum_{j=1}^{p} \beta_j^2 + \alpha \sum_{j=1}^{p} |\beta_j| \right]$ is the penalty term. λ is the regularization parameter, and α is the mixing parameter between L1 and L2 penalties.

For the 'glmnet' package the parameter α controls the balance between Lasso (L1 penalty) and Ridge (L2 penalty). When $\alpha = 1$, it's Lasso regression. When $\alpha = 0$, it's Ridge regression. For $0 < \alpha < 1$, it's Elastic Net regression, which combines both L1 and L2 penalties. In our case $\alpha = 1$ and we are using Lasso regression. [1]

For λ selection the dataset is divided into 'k' subsets, and the Lasso model is trained on 'k-1' of these subsets and validated on the remaining one, cycling through all 'k' subsets and averaging the validation errors to select the best lambda.

S3 Population and individual model fits

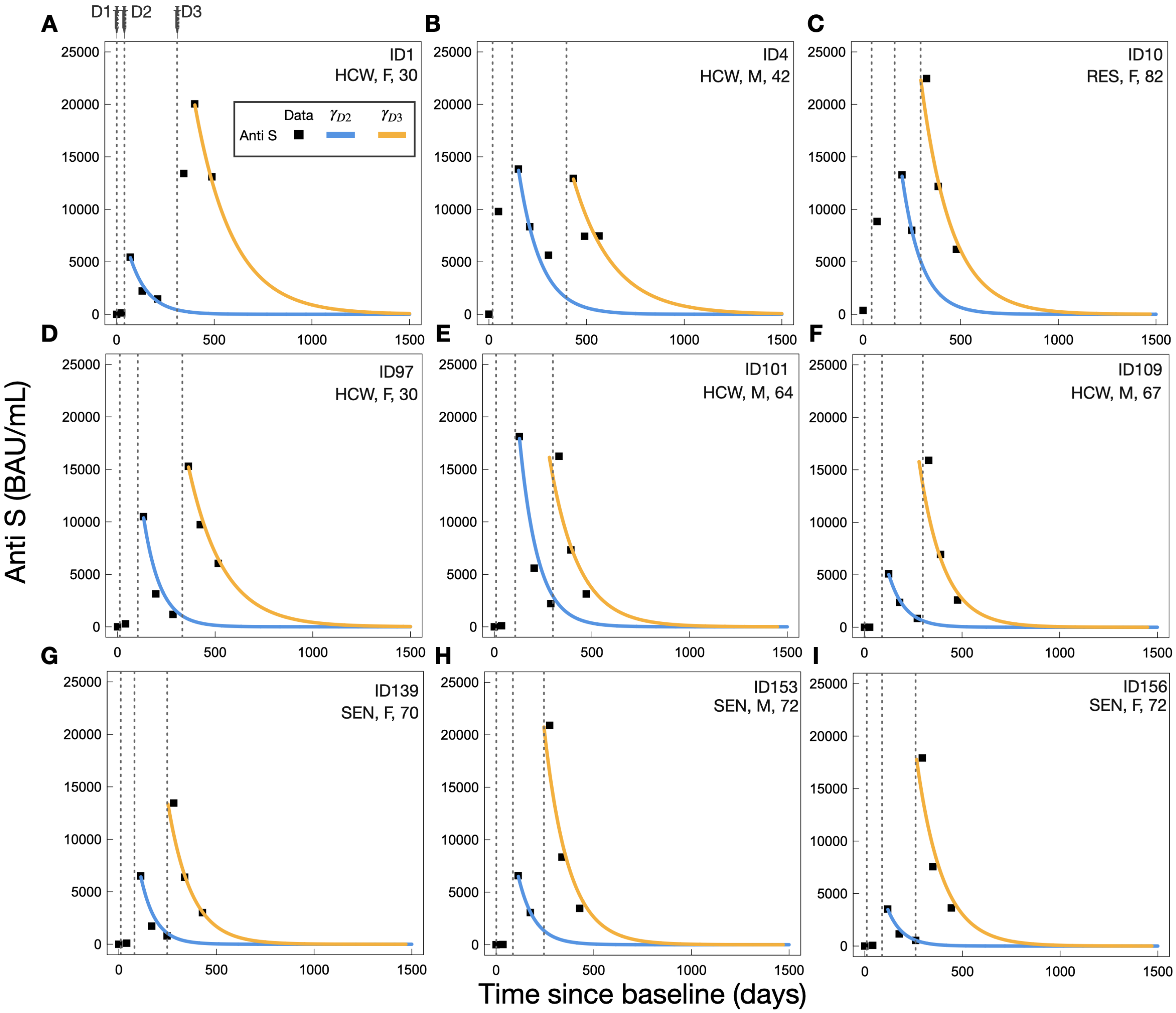

Figure S3: Example individual fits from the primary series and booster dose Anti-S trajectory data. Vertical dashed lines from left to right indicate day of SARS-CoV-2 dose one, two, and three, respectively.

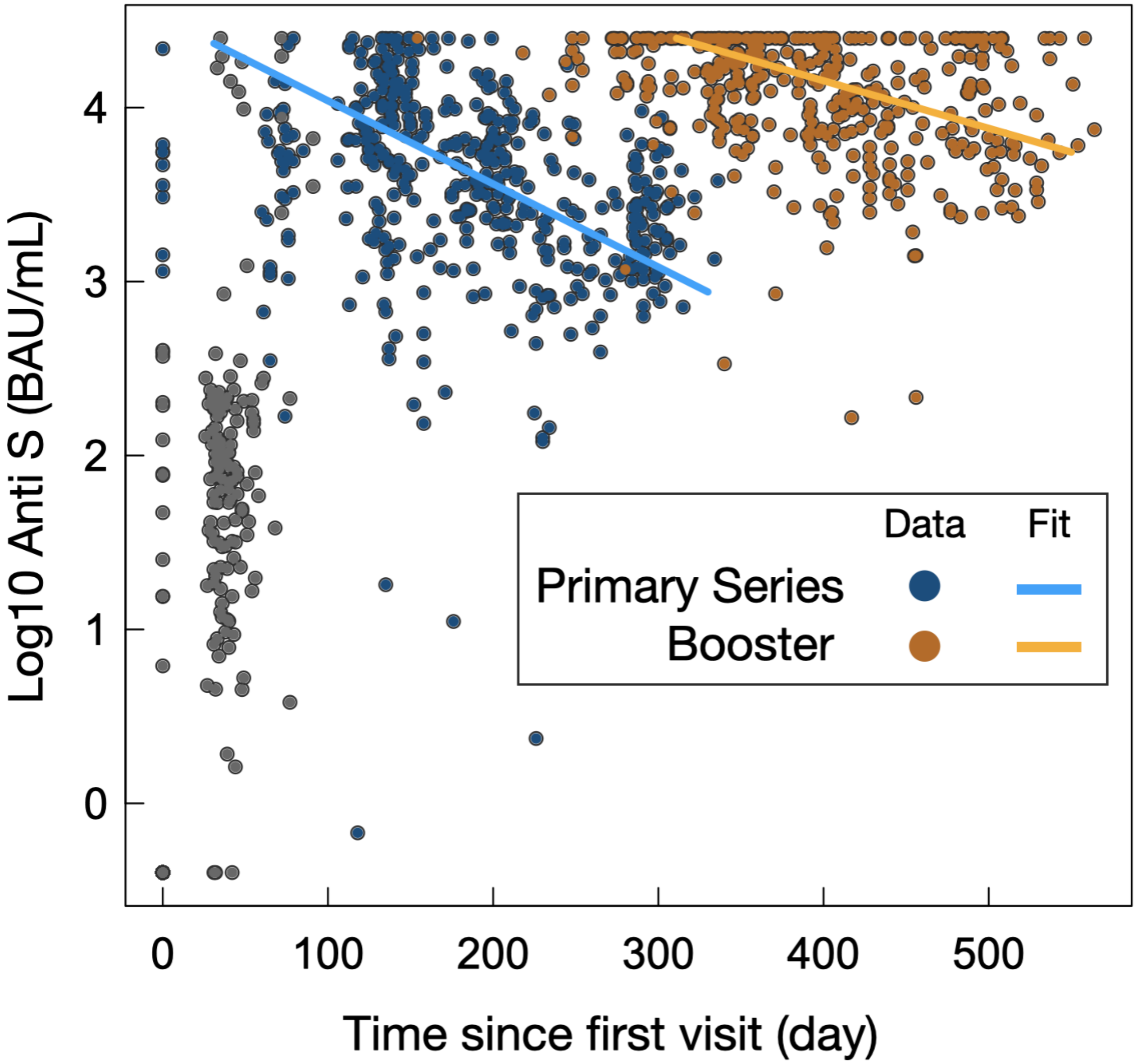

Figure S4: Population fits to all primary series and booster dose data.

S4 Exploring the age cutoff for statistical comparisons

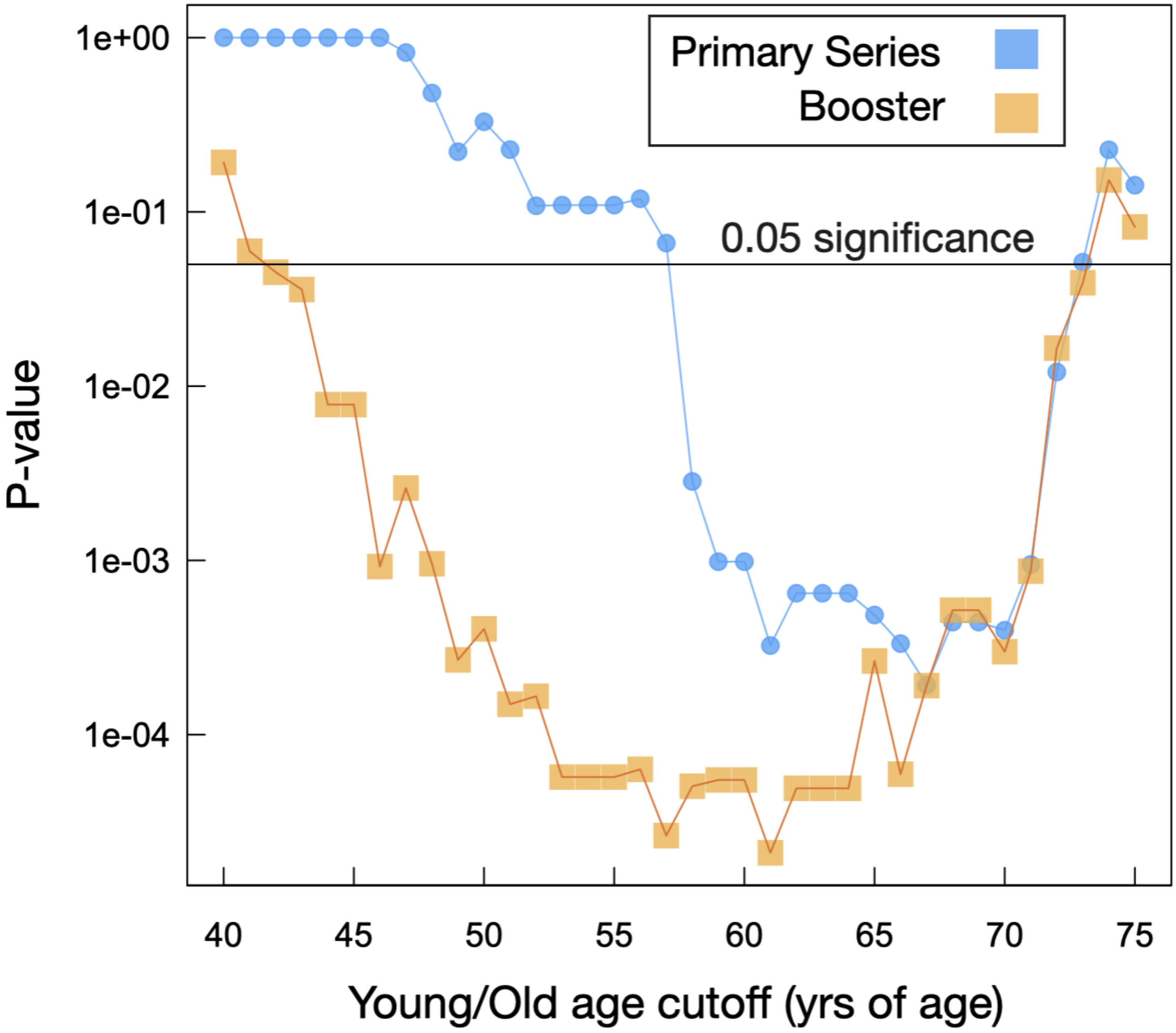

Figure S5: Bonferoni-corrected P-values as a function of young/old age cutoff.

S5 Multivariate statistical analysis

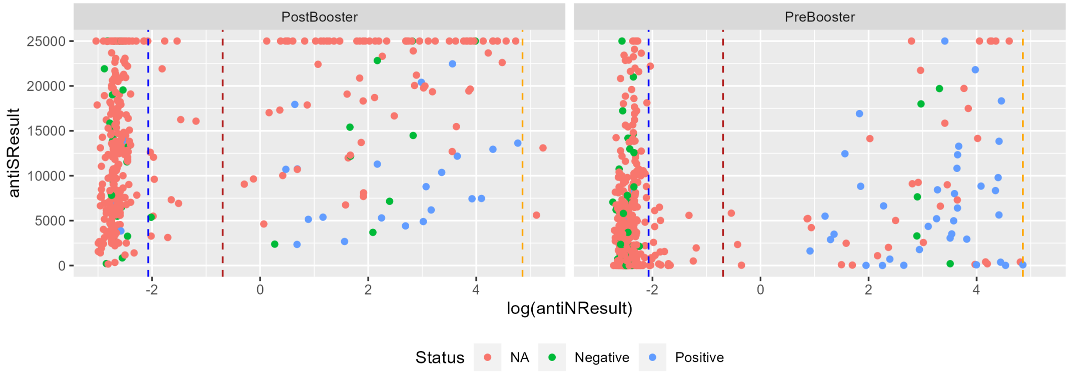

Figure S6: This figure illustrates the relationship between the logarithm of N-terminal domain (anti-N) antibody levels and spike protein (anti-S) antibody levels, with data points colored by COVID-19 test status. Vertical dashed lines represent thresholds used to infer assumed positive status before booster administration: low (dark red), alternative (light red), and high (green). These thresholds are determined by: Alternative Threshold (light red): Represented by the middle vertical dashed line. An arbitrary fixed value, An arbitrary fixed value, capturing the visual nadir in the distribution of anti-N results. Low Threshold (dark red): A value that's set at twice the lowest N-terminal domain antibody levels observed among those who tested positive for COVID-19. High Threshold (green color): Set by the highest N-terminal domain antibody levels observed among those who have not tested positive for COVID-19. Panel A and B represent pre- or post-booster dose data points, respectively.

	dose2Mu	dose3Mu	normDose2Mu	normDose3Mu	antiNResult	antiSCensor	Type shortHCW	Type shortRES	Type shortSEN	groupTypeNonHCW	Sex at BirthMale	assumedPositive	assumedPositiveInd0	assumedPositivePreBoosterInd1	ageMinMaxNormalized	Chronic neuroDisorder1	Hypertension1	Asthma1	Chronic lungDisease1	Chronic kidneyDisease1	Chronic heartDisease1	Chronic bloodDisorder1	Cancer1	Diabetes1
dose2Mu	1	0.77	1	0.77	-0.44	-0.32	-0.57	0.66	0.29	0.57	-0.24	-0.33	0.33	-0.2	0.52	0.77	0.45	-0.17	0.76	0.46	0.66	0.15	0.39	0.6
dose3Mu	0.77	1	0.77	1	-0.57	-0.55	-0.55	0.56	0.32	0.55	-0.24	-0.5	0.5	-0.25	0.55	0.66	0.55	-0.25	0.79	0.41	0.55	0.49	0.44	0.55
normalizedDose2Mu	1	0.77	1	0.77	-0.44	-0.32	-0.57	0.66	0.29	0.57	-0.24	-0.33	0.33	-0.2	0.52	0.77	0.45	-0.17	0.76	0.46	0.66	0.15	0.39	0.6
normalizedDose3Mu	0.77	1	0.77	1	-0.57	-0.55	-0.55	0.56	0.32	0.55	-0.24	-0.5	0.5	-0.25	0.55	0.66	0.55	-0.25	0.79	0.41	0.55	0.49	0.44	0.55
antiNResult	-0.44	-0.57	-0.44	-0.57	1	0.5	0.44	-0.32	-0.34	-0.44	0.25	0.86	-0.86	0.54	-0.47	-0.42	-0.44	-0.03	-0.26	-0.25	-0.33	-0.23	-0.37	-0.4
antiSCensor	-0.32	-0.55	-0.32	-0.55	0.5	1	0.24	-0.3	-0.11	-0.24	0	0.58	-0.58	0.11	-0.28	-0.42	-0.36	0.1	-0.31	-0.22	-0.26	-0.2	-0.34	-0.28
Type shortHCW	-0.57	-0.55	-0.57	-0.55	0.44	0.24	1	-0.61	-0.86	-1	0.02	0.35	-0.35	0.14	-0.98	-0.58	-0.65	0	-0.47	-0.48	-0.62	-0.02	-0.53	-0.57
Type shortRESIDENT	0.66	0.56	0.66	0.56	-0.32	-0.3	-0.61	1	0.11	0.61	0.03	-0.16	0.16	0.04	0.62	0.84	0.63	-0.13	0.66	0.78	0.83	-0.1	0.39	0.76
Type shortSENIOR	0.29	0.32	0.29	0.32	-0.34	-0.11	-0.86	0.11	1	0.86	-0.04	-0.33	0.33	-0.19	0.83	0.18	0.41	0.09	0.16	0.1	0.24	0.09	0.41	0.22
groupTypenon-HCW	0.57	0.55	0.57	0.55	-0.44	-0.24	-1	0.61	0.86	1	-0.02	-0.35	0.35	-0.14	0.98	0.58	0.65	0	0.47	0.48	0.62	0.02	0.53	0.57
Sex at BirthMale	-0.24	-0.24	-0.24	-0.24	0.25	0	0.02	0.03	-0.04	-0.02	1	0.26	-0.26	0.29	0	-0.15	-0.03	0.02	-0.02	-0.02	0.05	-0.24	-0.31	-0.13
assumedPositivePositive	-0.33	-0.5	-0.33	-0.5	0.86	0.58	0.35	-0.16	-0.33	-0.35	0.26	1	-1	0.71	-0.37	-0.31	-0.39	0.06	-0.11	-0.03	-0.18	-0.26	-0.29	-0.27
assumedPositiveIndicator0	0.33	0.5	0.33	0.5	-0.86	-0.58	-0.35	0.16	0.33	0.35	-0.26	-1	1	-0.71	0.37	0.31	0.39	-0.06	0.11	0.03	0.18	0.26	0.29	0.27
assumedPositivePreBoosterIndicator1	-0.2	-0.25	-0.2	-0.25	0.54	0.11	0.14	0.04	-0.19	-0.14	0.29	0.71	-0.71	1	-0.15	-0.1	-0.23	0	0.05	0.13	-0.03	-0.21	-0.13	-0.13
ageMinMaxNormalized	0.52	0.55	0.52	0.55	-0.47	-0.28	-0.98	0.62	0.83	0.98	0	-0.37	0.37	-0.15	1	0.56	0.73	0.05	0.46	0.51	0.63	0	0.51	0.57
Chronic neurological disorder1	0.77	0.66	0.77	0.66	-0.42	-0.42	-0.58	0.84	0.18	0.58	-0.15	-0.31	0.31	-0.1	0.56	1	0.46	-0.23	0.68	0.57	0.71	-0.02	0.49	0.69
Hypertension1	0.45	0.55	0.45	0.55	-0.44	-0.36	-0.65	0.63	0.41	0.65	-0.03	-0.39	0.39	-0.23	0.73	0.46	1	0.07	0.47	0.48	0.58	0.12	0.28	0.44
Asthma1	-0.17	-0.25	-0.17	-0.25	-0.03	0.1	0	-0.13	0.09	0	0.02	0.06	-0.06	0	0.05	-0.23	0.07	1	-0.11	-0.02	0	-0.16	-0.19	-0.23
Chronic lung disease1	0.76	0.79	0.76	0.79	-0.26	-0.31	-0.47	0.66	0.16	0.47	-0.02	-0.11	0.11	0.05	0.46	0.68	0.47	-0.11	1	0.54	0.69	0.34	0.29	0.48
Chronic kidney disease1	0.46	0.41	0.46	0.41	-0.25	-0.22	-0.48	0.78	0.1	0.48	-0.02	-0.03	0.03	0.13	0.51	0.57	0.48	-0.02	0.54	1	0.75	-0.14	0.5	0.63
Chronic heart disease1	0.66	0.55	0.66	0.55	-0.33	-0.26	-0.62	0.83	0.24	0.62	0.05	-0.18	0.18	-0.03	0.63	0.71	0.58	0	0.69	0.75	1	-0.1	0.3	0.83
Chronic blood disorder1	0.15	0.49	0.15	0.49	-0.23	-0.2	-0.02	-0.1	0.09	0.02	-0.24	-0.26	0.26	-0.21	0	-0.02	0.12	-0.16	0.34	-0.14	-0.1	1	-0.03	-0.09
Cancer1	0.39	0.44	0.39	0.44	-0.37	-0.34	-0.53	0.39	0.41	0.53	-0.31	-0.29	0.29	-0.13	0.51	0.49	0.28	-0.19	0.29	0.5	0.3	-0.03	1	0.42
Diabetes1	0.6	0.55	0.6	0.55	-0.4	-0.28	-0.57	0.76	0.22	0.57	-0.13	-0.27	0.27	-0.13	0.57	0.69	0.44	-0.23	0.48	0.63	0.83	-0.09	0.42	1

Table S1: Correlation relationships between various chronic commodities, demographic factors.

term	estimate	std.error	statistic	p.value	model	conf.low	conf.high	signif.star
Anti-N Result	-0.000005	0.000003	-1.717728	0.086289	Primary Series	-0.000010	0.000001	
Resident (HCW)	0.000915	0.000149	6.135707	0.000000	Primary Series	0.000623	0.001207	***
Senior (HCW)	0.000381	0.000102	3.731346	0.000206	Primary Series	0.000181	0.000580	***
Sex Male (Female)	-0.000214	0.000095	-2.255650	0.024401	Primary Series	-0.000400	-0.000028	*
Chronic Neurological Disorder	0.001029	0.000188	5.476516	0.000000	Primary Series	0.000661	0.001398	***
Chronic Lung Disease	0.001873	0.000221	8.468033	0.000000	Primary Series	0.001439	0.002306	***
Cancer	0.000150	0.000235	0.636438	0.524699	Primary Series	-0.000311	0.000610	
predicted infection (pre booster)	-0.000419	0.000143	-2.921897	0.003591	Primary Series	-0.000699	-0.000138	**
Anti-N Result	0.000007	0.000003	2.117883	0.034572	Booster Dose	0.000001	0.000014	*
Anti-Spike Censored	-0.000856	0.000184	-4.652644	0.000004	Booster Dose	-0.001216	-0.000495	***
Resident (HCW)	0.000175	0.000247	0.707532	0.479494	Booster Dose	-0.000310	0.000660	
Senior (HCW)	0.000002	0.000196	0.010361	0.991737	Booster Dose	-0.000382	0.000386	
Sex Male (Female)	-0.000367	0.000108	-3.401200	0.000713	Booster Dose	-0.000579	-0.000156	***
Min-Max Normalized Age	0.000846	0.000356	2.376604	0.017767	Booster Dose	0.000148	0.001544	*
Hypertension	0.000325	0.000136	2.392787	0.017010	Booster Dose	0.000059	0.000591	*
Asthma	-0.000783	0.000179	-4.369616	0.000015	Booster Dose	-0.001134	-0.000432	***
Chronic Lung Disease	0.004135	0.000275	15.019343	0.000000	Booster Dose	0.003595	0.004675	***
Cancer	0.000494	0.000255	1.938031	0.053061	Booster Dose	-0.000006	0.000994	
predicted infection (any phase)	-0.000583	0.000176	-3.315878	0.000965	Booster Dose	-0.000928	-0.000239	***

Table S2: Multivariate Linear Regression Coefficients and Significance

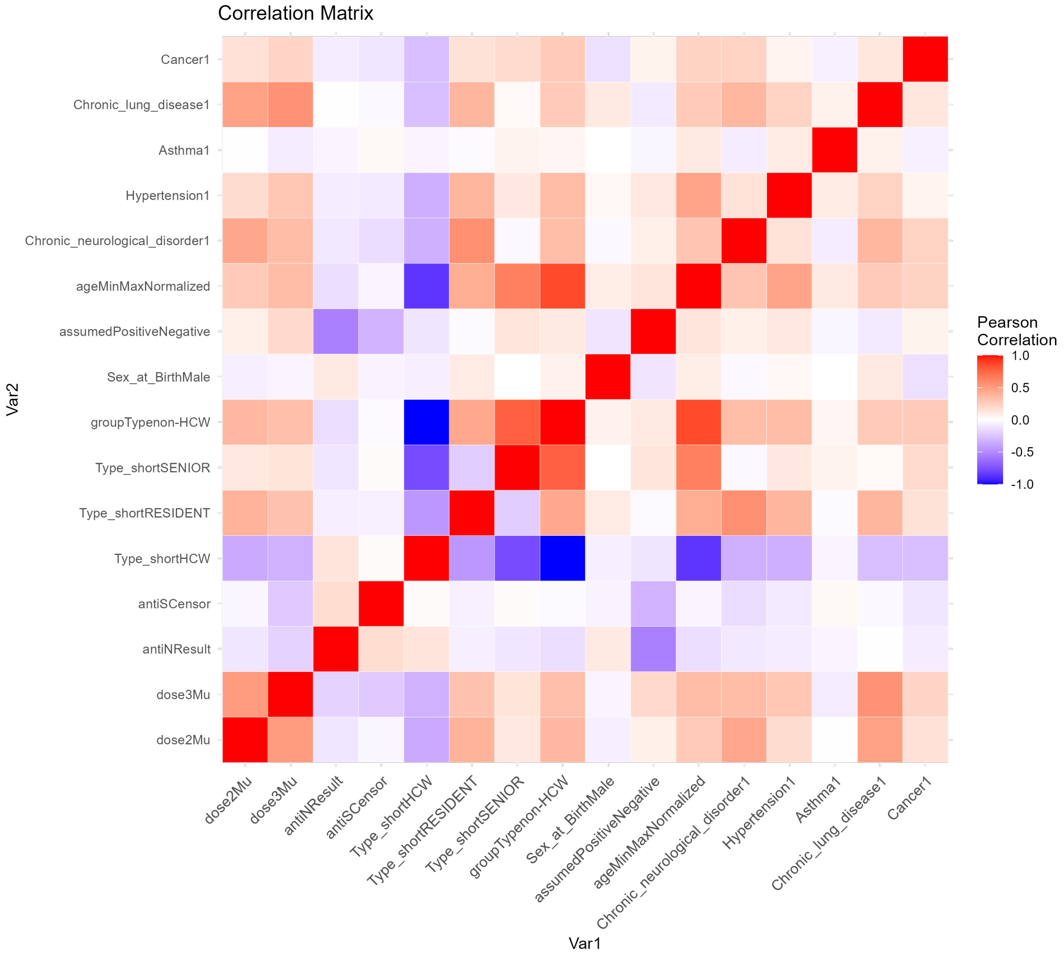

Figure S7: Correlation heatmap illustrating relationships between various chronic commodities, demographic factors, and vaccine dose responses available in the data. Red indicates positive correlation, blue indicates negative correlation, and white indicates no correlation.

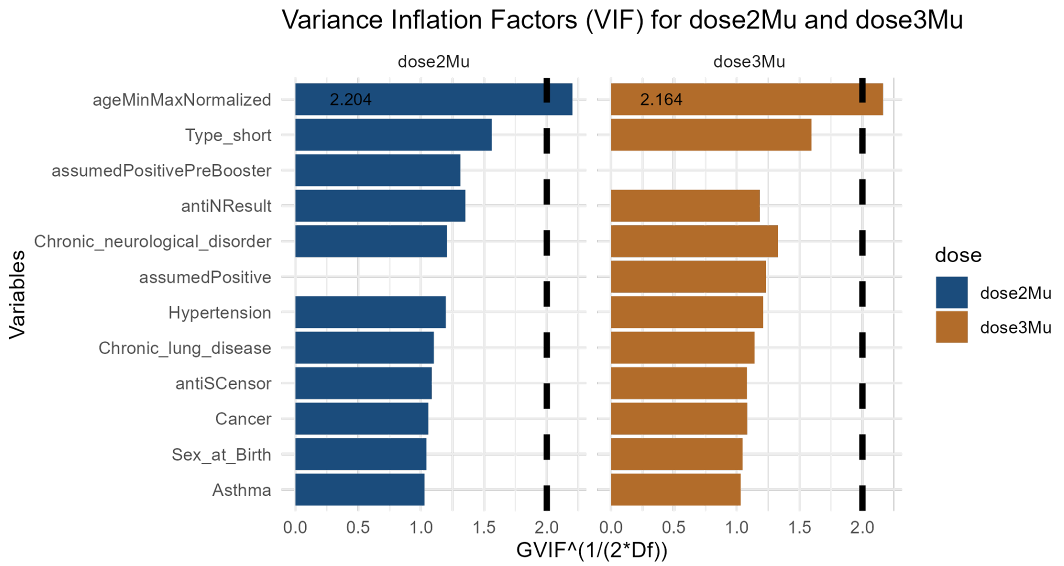

Figure S8: Variance Inflation Factor (VIF) Analysis. The figure displays GVIF$^{(1/(2*Df))}$ values for each variable considerd in the regression models. All variables, except for ‘ageMinMaxNormalized‘, exhibit values below the threshold of 2. This suggests that there are no substantial multicollinearity concerns within these models, with the exception of the ‘ageMinMaxNormalized‘ variable. In the VIF analysis, the variable ‘ageMinMaxNormalized‘ exhibited a marginally high GVIF value (2.20 for ‘dose2Mu‘ and 2.16 for ‘dose3Mu‘), just slightly above the conventional threshold of 2 [5–7]. This prompts a discussion on whether to include or exclude this variable from the model. The slight exceedance of the threshold does not necessarily denote a substantial violation of the assumptions underlying the model. The variable ‘ageMinMaxNormalized‘ is the focus of our analysis and excluding it based solely on a marginally high VIF value could lead to an incomplete or biased understanding of the underlying relationships. For this reason we include ‘ageMinMaxNormalized‘ in the analysis.

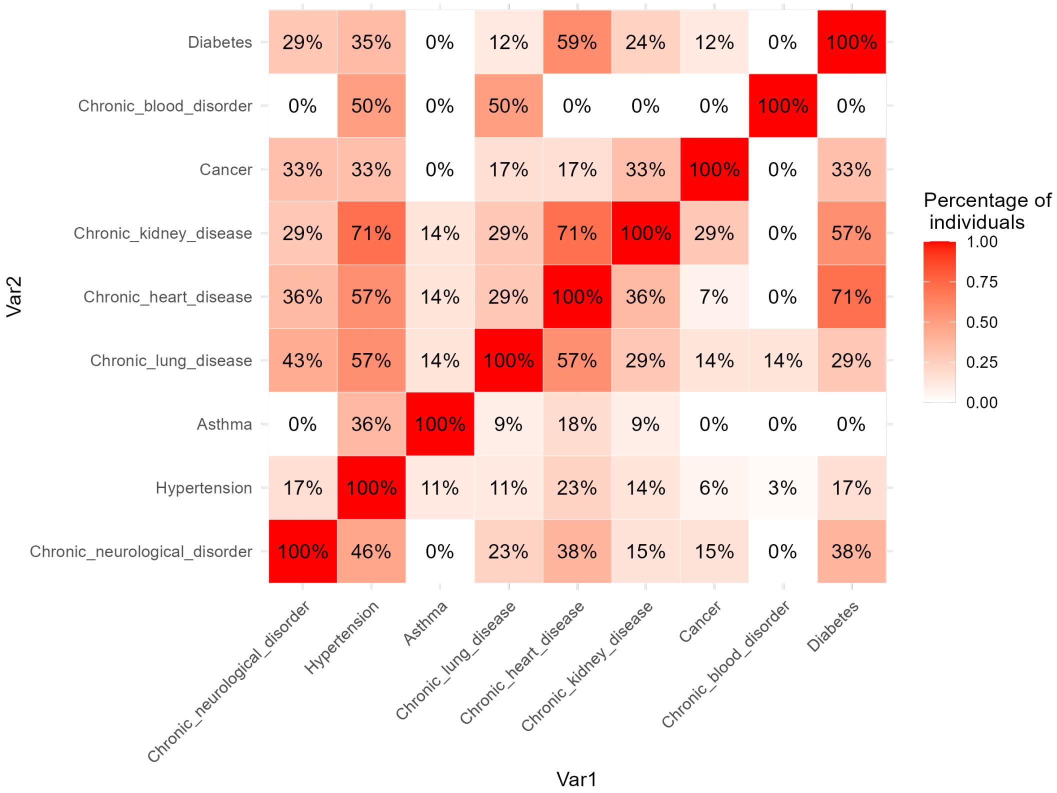

Figure S9: The heatmap portrays the percentage of individuals in our dataset with co-existing chronic diseases. Each cell represents the proportion of individuals having a specific condition (represented by columns) given the presence of another condition (indicated by rows). The color gradient corresponds with the percentage: deeper red shades signify a higher percentage, while white denotes a lower one. This represents the conditional probability, in percentage terms, that an individual possesses the comorbidity listed in the row, given they have the one in the column. For example, if someone has Hypertension (row), the probability they also have Hypertension (column) is 100%. However, the probability that they have Diabetes (column) drops to 17%. The repetition of percentages across different conditions can be attributed to the limited count of individuals with certain diseases in the dataset.

References

[1] J. Friedman, T. Hastie, R. Tibshirani, *et al.*, *glmnet: Lasso and Elastic-Net Regularized Generalized Linear Models*, 2023. R package version 4.1-8.

[2] J. Friedman, T. Hastie, and R. Tibshirani, "Regularization paths for generalized linear models via coordinate descent," *Journal of Statistical Software*, vol. 33, no. 1, p. 1, 2010.

[3] N. Simon, J. Friedman, T. Hastie, and R. Tibshirani, "Regularization paths for cox's proportional hazards model via coordinate descent," *Journal of Statistical Software*, vol. 39, no. 5, p. 1, 2011.

[4] R. Tibshirani, J. Bien, J. Friedman, T. Hastie, N. Simon, J. Taylor, and R. J. Tibshirani, "Strong rules for discarding predictors in lasso-type problems," *Journal of the Royal Statistical Society: Series B (Statistical Methodology)*, vol. 74, no. 2, pp. 245–266, 2012.

[5] R. M. O'Brien, "A caution regarding rules of thumb for variance inflation factors," *Quality & Quantity*, vol. 41, no. 5, pp. 673–690, 2007.

[6] J. F. Hair, *Multivariate Data Analysis.* Prentice Hall, 7 ed., 2010.

[7] A. F. Zuur, E. N. Ieno, and C. S. Elphick, "A protocol for data exploration to avoid common statistical problems," *Methods in Ecology and Evolution*, vol. 1, no. 1, pp. 3–14, 2010.
